# Antimicrobial Resistance Trends in Carbapenem-Resistant Enterobacterales in a Saudi Healthcare Setting

**DOI:** 10.3390/microorganisms14081604

**Published:** 2026-07-23

**Authors:** Wael A. Alghamdi, Khalid M. Orayj, Abdullah M. Alshehri, Saeed A. Alqahtani, Ahmed R. N. Ibrahim

**Affiliations:** 1 Department of Clinical Pharmacy, College of Pharmacy, King Khalid University, Abha 62223, Saudi Arabia; 2 Department of Pharmacy, Khamis Mushait General Hospital, Khamis Mushait 62433, Saudi Arabia; 3 Faculty of Pharmacy, Minia University, Minia 61519, Egypt

**Keywords:** carbapenem-resistant Enterobacterales, *Klebsiella pneumoniae*: antimicrobial resistance, antimicrobial stewardship, Saudi Arabia

## Abstract

Longitudinal data on carbapenem-resistant Enterobacterales (CRE) from Saudi secondary healthcare settings remain limited. This study evaluated eight-year antimicrobial resistance trends among CRE isolates to provide locally relevant evidence for antimicrobial stewardship and empiric therapy. This retrospective surveillance study analyzed Enterobacterales isolates recovered from clinical specimens between 2017 and 2024 from a Saudi healthcare setting. Isolates were deduplicated by including only the first isolate per patient per year. CRE were defined as Enterobacterales demonstrating non-susceptibility to imipenem or meropenem. Demographic data, specimen sources, species distribution, and antimicrobial resistance profiles were summarized descriptively. Temporal trends in CRE prevalence and antimicrobial non-susceptibility were evaluated using logistic regression. Factors associated with CRE were assessed using multivariable logistic regression analyses. A total of 2378 Enterobacterales isolates were identified, of which 684 (28.8%) were CRE. CRE prevalence increased significantly over the study period, rising from 9.5% in 2017 to a peak of 40.1% in 2021 before declining to 27.1% in 2024. Multivariable logistic regression demonstrated that collection year (adjusted OR 1.12, 95% CI 1.08–1.17; *p* < 0.0001), male sex, and specimen source were independently associated with CRE. *Klebsiella pneumoniae* was the predominant species. High non-susceptibility rates were observed for cephalosporins and fluoroquinolones, while aminoglycosides and tigecycline demonstrated comparatively lower resistance. Significant temporal trends in non-susceptibility were observed for cefepime, ceftazidime, ciprofloxacin, and tigecycline. CRE represented a substantial proportion of Enterobacterales isolates and demonstrated extensive antimicrobial resistance. These findings highlight the importance of continuous local surveillance to inform empirical antimicrobial therapy, support antimicrobial stewardship, and monitor evolving resistance patterns.

## 1. Introduction

Carbapenem-resistant Enterobacterales (CRE) have emerged as a major global public health threat and are associated with substantial therapeutic and infection control challenges. Enterobacterales, including *Klebsiella pneumoniae*, *Escherichia coli*, and *Enterobacter* spp., are among the leading causes of healthcare-associated infections such as bloodstream infections, pneumonia, urinary tract infections, and intra-abdominal infections. Their capacity to spread rapidly within healthcare environments, together with frequent co-resistance to multiple non-carbapenem antimicrobial classes, further complicates infection control and treatment. When carbapenem resistance develops, treatment options become limited and clinical outcomes worsen, with reported increases in length of hospital stay, healthcare costs, and mortality [1,2].

The growing burden of CRE has been linked to the widespread use of broad-spectrum antibiotics, particularly carbapenems, in the treatment of infections caused by multidrug-resistant Gram-negative organisms. Because of their clinical impact and limited treatment options, CRE have been classified by the World Health Organization among the highest-priority pathogens for research and development of new antimicrobial agents [3,4]. The global burden of CRE has increased substantially over the past decade, with marked geographic variation in prevalence and carbapenemase distribution. High endemicity has been reported in parts of Southern Europe, Latin America, and Asia, while increasing incidence has also been documented in North America and other regions. The dissemination of major carbapenemases, including KPC, NDM, OXA-48-like, VIM, and IMP enzymes, has contributed to the worldwide spread of CRE and represents a major challenge for infection prevention, antimicrobial stewardship, and the development of effective treatment strategies [1,3,5].

In Saudi Arabia and the broader Gulf region, CRE have become an increasing concern in hospital settings. A recent systematic review and meta-analysis from Saudi Arabia reported pooled carbapenem resistance estimates of 6.6% for imipenem, 9.1% for meropenem, and 18.6% for ertapenem, with substantial heterogeneity across studies and regions [6]. Regional studies have consistently identified *K. pneumoniae* as the predominant CRE species, with OXA-48-like and NDM carbapenemases among the most frequently reported resistance mechanisms in the Arabian Peninsula [7,8]. Reports from Saudi Arabia have documented increasing CRE prevalence and considerable variability in resistance burden between institutions [7,9]. Surveillance data from western Saudi Arabia showed increasing CRE prevalence across tertiary care hospitals, with marked inter-facility variation, highlighting the importance of local epidemiological monitoring [9]. Similar observations have been reported in the United Arab Emirates, where national surveillance data demonstrated temporal changes in CRE prevalence across multiple healthcare facilities [10]. These findings highlight the importance of local epidemiological studies because resistance patterns and carbapenemase epidemiology may differ substantially between healthcare settings.

Despite increasing recognition of CRE as a major healthcare threat in Saudi Arabia, long-term epidemiological data from some regions remain limited, particularly outside large tertiary centers. Continuous local surveillance is essential to monitor temporal trends, define species distribution, and support antimicrobial stewardship and infection prevention strategies. Therefore, this study aimed to evaluate CRE prevalence, species distribution, antimicrobial resistance patterns, temporal trends, and factors associated with CRE over an eight-year period in a Saudi secondary healthcare setting.

## 2. Materials and Methods

### 2.1. Study Design

This retrospective surveillance study evaluated antimicrobial resistance patterns among Enterobacterales isolates recovered from hospitalized patients at a secondary healthcare setting in the Aseer region of Saudi Arabia. Clinical microbiology laboratory records from January 2017 through December 2024 were reviewed to determine the prevalence, species distribution, antimicrobial resistance profiles, and temporal trends of CRE. The unit of analysis was the deduplicated patient-year isolate, allowing longitudinal assessment of resistance patterns while reducing the influence of repeated cultures from the same patient. The study was approved by the Aseer Institutional Review Board (approval number F10-2-2025). All patient data were de-identified prior to analysis to ensure confidentiality.

### 2.2. Data Collection

All clinical Enterobacterales isolates recovered during the study period were screened for inclusion. To minimize bias from repeated sampling, isolates were deduplicated at the patient level. Only the first isolate per patient per calendar year was included in the analysis. Accordingly, each included isolate represented a unique patient within a given calendar year, although the same patient could contribute one isolate in different years. When multiple specimens were collected from the same patient on the same day, a hierarchy of clinical significance was applied. Isolates from blood and sterile body fluids were prioritized, followed by respiratory specimens, urine samples, wound or tissue specimens, and stool samples. Subsequent isolates from the same patient within the same year were excluded regardless of changes in antimicrobial susceptibility profiles. Because this was a laboratory-based surveillance study, isolates could represent infection or colonization.

Available data extracted from laboratory and hospital information systems included patient age, sex, nationality, and specimen source. Age was categorized into predefined groups for descriptive and regression analyses (0–18, 19–30, 31–45, 46–60, and >60 years). Specimen sources were categorized into five groups: blood and sterile body fluids, respiratory tract, urine and genital tract, wound, pus, and tissue, and stool and gastrointestinal tract specimens.

### 2.3. Laboratory Procedures and Susceptibility Interpretation

Clinical specimens were processed in the microbiology laboratory according to routine standard operating procedures for bacterial culture. Specimens were inoculated onto appropriate culture media based on specimen type and incubated under standard aerobic conditions. Bacterial isolates were identified using standard culture methods followed by automated identification with the MicroScan^®^ system (Beckman Coulter, Brea, CA, USA). Antimicrobial susceptibility testing was performed using the same automated system. No molecular identification or molecular resistance testing was performed. Susceptibility results were interpreted according to the Clinical and Laboratory Standards Institute (CLSI) breakpoints applicable at the time of testing [11]. For analytical purposes, isolates categorized as intermediate or resistant were considered non-susceptible. CRE were defined as Enterobacterales isolates demonstrating non-susceptibility to at least one carbapenem agent (imipenem or meropenem) according to CLSI interpretive criteria. Ertapenem susceptibility was not routinely performed in the study laboratory during the surveillance period and was therefore not included in the case definition.

### 2.4. Data Analysis

Annual CRE prevalence was calculated as the proportion of CRE isolates among all Enterobacterales isolates for each study year. Species distribution and antimicrobial non-susceptibility rates were summarized descriptively. Temporal trends in overall CRE prevalence and antimicrobial non-susceptibility for individual antimicrobial agents were evaluated using logistic regression with calendar year entered as a continuous predictor. Factors independently associated with CRE were assessed using multivariable logistic regression, with CRE status as the dependent variable and collection year, age group, sex, and specimen source included as independent variables. Stool and gastrointestinal specimens were excluded from the multivariable model because of the small number of isolates. Results are presented as odds ratios (ORs) with 95% confidence intervals (CIs). Statistical analyses were performed using JMP Pro, version 18 (SAS Institute Inc., Cary, NC, USA). All tests were two-sided, and statistical significance was defined as *p* < 0.05.

## 3. Results

### 3.1. Demographic Characteristics

During the eight-year surveillance period, 2378 non-duplicate Enterobacterales isolates, each representing a unique patient within a calendar year, were identified (Table 1). Among these, 684 isolates (28.8%) met the criteria for CRE. Isolates were more frequently recovered from male patients (58.1%), and more than half were obtained from patients older than 60 years (50.7%). Urine and genital tract specimens represented the largest proportion of isolates (41.8%), followed by respiratory tract (22.7%) and wound, pus, or tissue specimens (22.6%). Blood and sterile body fluid isolates accounted for 12.6% of specimens, while gastrointestinal isolates were uncommon (0.3%).

### 3.2. Factors Associated with CRE

Multivariable logistic regression identified collection year, male sex, age group, and specimen source as factors associated with CRE (Table 2). Each additional calendar year was associated with a 12% increase in the adjusted odds of CRE (adjusted OR 1.12, 95% CI 1.08–1.17; *p* < 0.0001). Male patients had 48% higher adjusted odds of CRE than female patients (adjusted OR 1.48, 95% CI 1.21–1.81; *p* < 0.0001). Patients aged 31–45 years had lower adjusted odds than those aged > 60 years (adjusted OR 0.70, 95% CI 0.54–0.90; *p* = 0.0056), whereas the other age groups were not significantly different from the reference category. Compared with blood and sterile body fluid specimens, respiratory specimens were associated with higher adjusted odds of CRE (adjusted OR 2.05, 95% CI 1.51–2.79; *p* < 0.0001), whereas urine and genital tract specimens were associated with lower adjusted odds (adjusted OR 0.65, 95% CI 0.49–0.88; *p* = 0.0048).

### 3.3. Temporal Trend in CRE Prevalence

**Figure 1:**
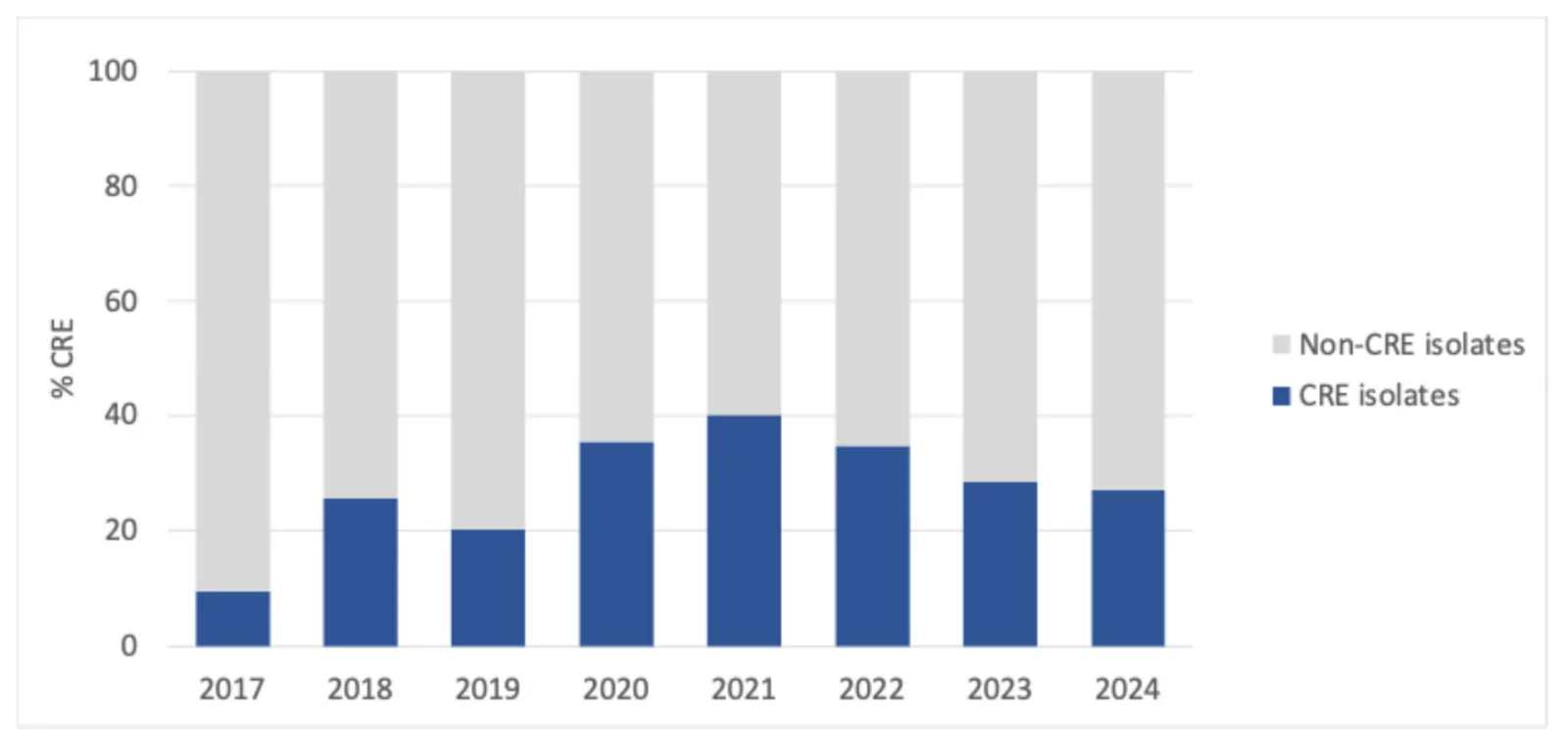
Annual proportion of CRE isolates, 2017–2024.

The prevalence of CRE among Enterobacterales increased significantly over the study period (Figure 1; Table 2). Each additional calendar year was associated with a 12% increase in the adjusted odds of CRE (adjusted OR 1.12, 95% CI 1.08–1.17; *p* < 0.0001). CRE prevalence was 9.5% in 2017, increased over subsequent years to a peak of 40.1% in 2021, and then declined gradually to 27.1% by 2024, while remaining higher than at the beginning of the surveillance period.

### 3.4. Antimicrobial Resistance Profile of CRE Isolates

**Figure 2:**
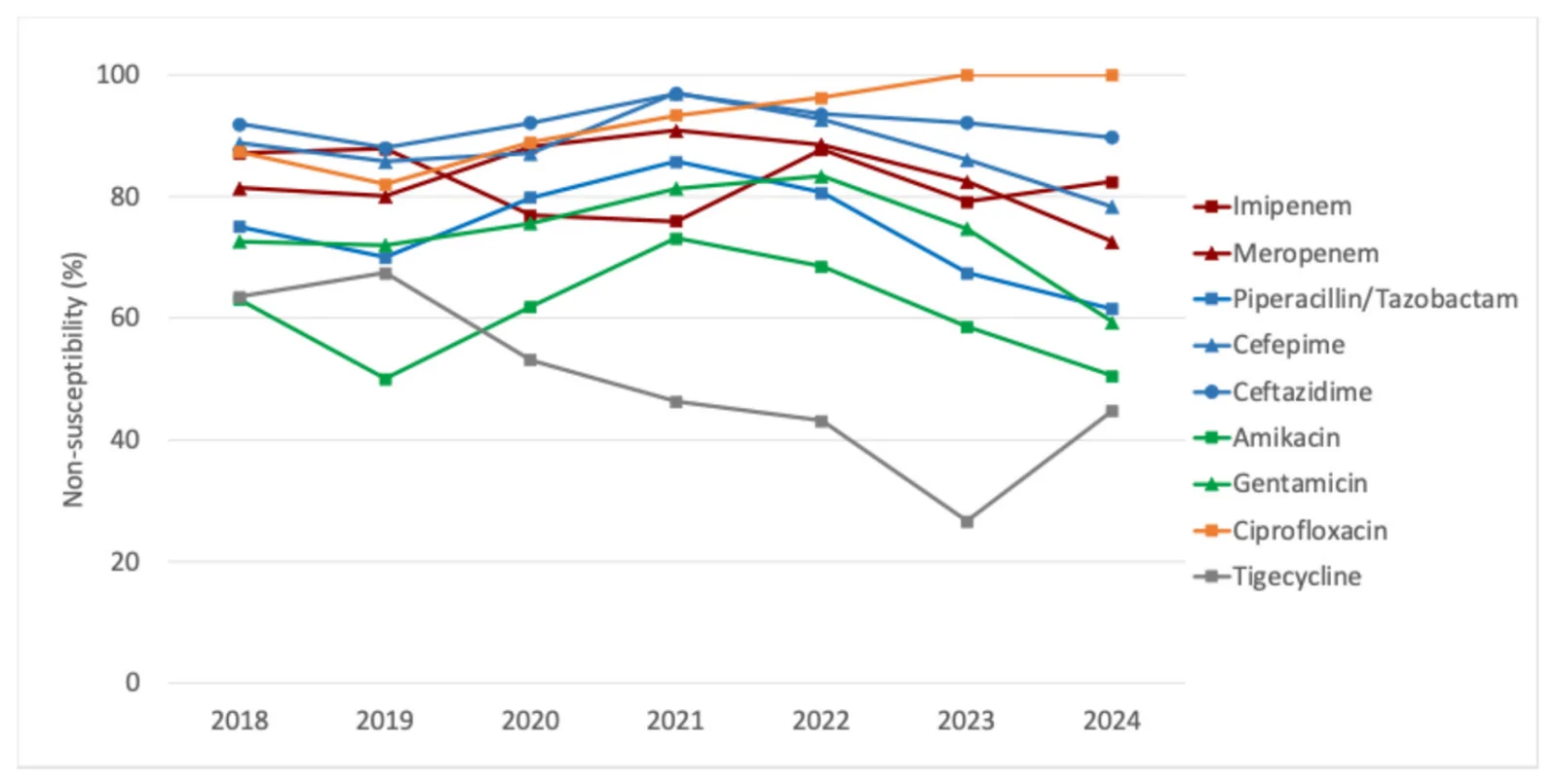
Trends in antimicrobial non-susceptibility among CRE isolates, 2018–2024. Lines represent the percentage of CRE isolates categorized as non-susceptible (intermediate or resistant) to each antimicrobial agent. The graph was limited to this period due to the small number of isolates prior to 2018.

Non-susceptibility to carbapenems exceeded 75% annually for imipenem, while meropenem non-susceptibility peaked at 90.8% in 2021 before declining to 72.5% in 2024 (Table 3; Figure 2). Among β-lactam agents, piperacillin/tazobactam non-susceptibility increased from 50.0% in 2017 to a peak of 85.7% in 2021, followed by a decline to 61.5% in 2024. Non-susceptibility to cefepime and ceftazidime also reached its highest levels in 2021 (97.0% and 96.9%, respectively) before decreasing modestly in subsequent years. Fluoroquinolone resistance was particularly high, with ciprofloxacin non-susceptibility increasing over time and reaching 100% in both 2023 and 2024. Among aminoglycosides, amikacin demonstrated comparatively lower resistance, with non-susceptibility peaking at 73.1% in 2021 and declining to 50.5% in 2024. Gentamicin non-susceptibility reached its highest level in 2022 (83.3%) before decreasing to 59.3% by 2024. Tigecycline demonstrated the lowest overall non-susceptibility rates among the antimicrobials evaluated. Resistance increased to 67.4% in 2019, declined steadily to 26.6% in 2023, and increased modestly to 44.8% in 2024. Temporal trend analysis demonstrated significant changes in non-susceptibility for cefepime (*p* = 0.0035), ceftazidime (*p* < 0.0001), ciprofloxacin (*p* < 0.0001), and tigecycline (*p* = 0.0063), whereas no significant trends were observed for the remaining antimicrobial agents (Table 3).

**Figure 3:**
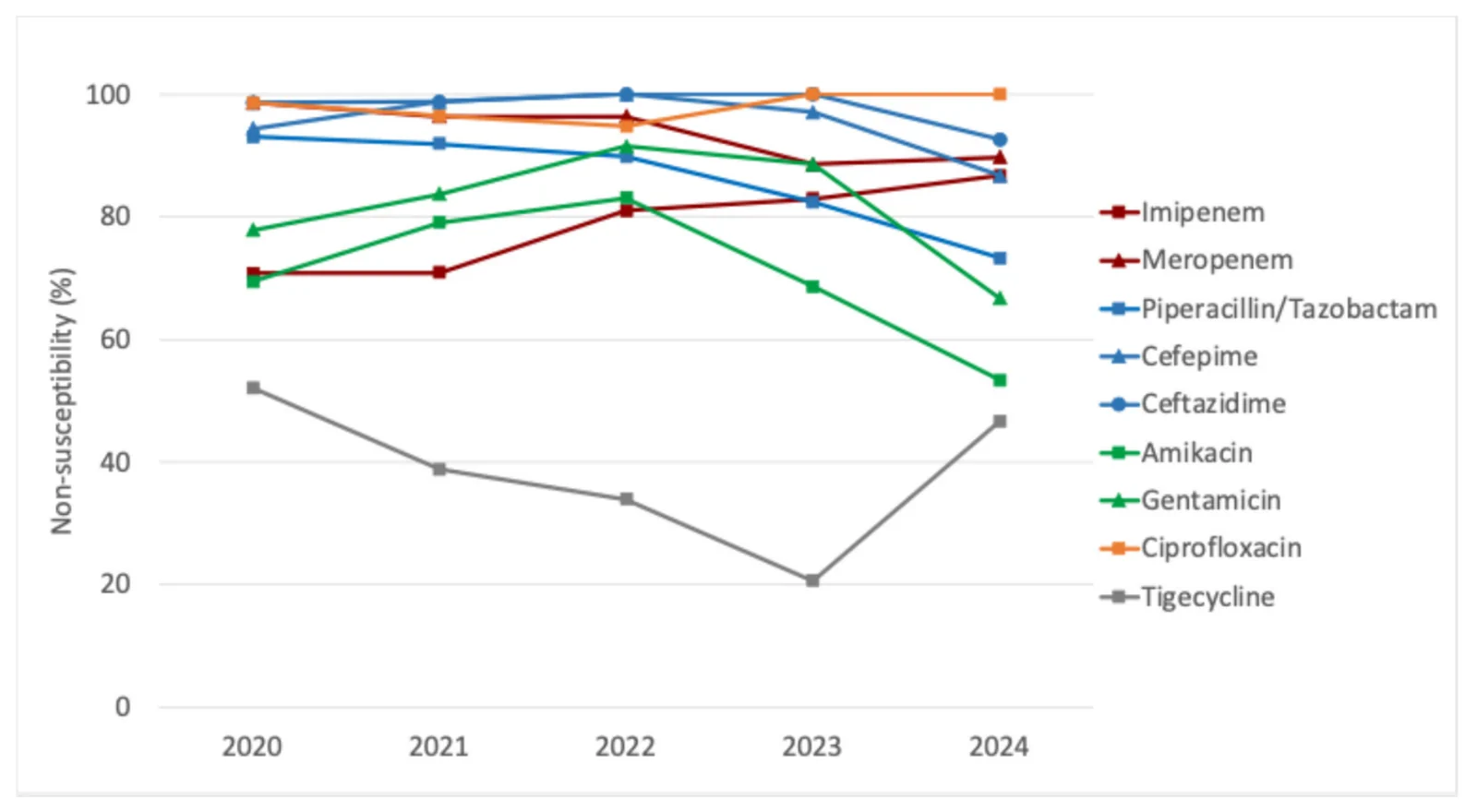
Trends in antimicrobial non-susceptibility among carbapenem-resistant *K. pneumoniae* isolates, 2020–2024. Lines represent the percentage of carbapenem-resistant *K. pneumoniae* isolates categorized as non-susceptible (intermediate or resistant) to each antimicrobial agent. The graph was limited to 2020–2024 because earlier years had fewer carbapenem-resistant *K. pneumoniae* isolates and more variable antimicrobial-specific testing denominators.

Resistance patterns among carbapenem-resistant *K. pneumoniae* were broadly similar to those observed in the overall CRE population (Figure 3). Species-specific antimicrobial non-susceptibility rates for the three predominant CRE species are presented in Supplementary Table S1. Non-susceptibility to carbapenems, cephalosporins, and fluoroquinolones remained high throughout the study period, whereas aminoglycosides demonstrated comparatively lower non-susceptibility and tigecycline retained the greatest activity, although resistance levels fluctuated across years.

### 3.5. Species Composition of CRE Isolates

The species distribution of CRE isolates varied over time (Table 4). *K. pneumoniae* was the predominant CRE species throughout the study period, accounting for 33.3–64.2% of annual CRE isolates and reaching its highest count in 2021 (n = 86). *E. coli* was the second most common CRE species and increased in the later years, accounting for 30.4% of CRE isolates in 2024 (n = 28). *Enterobacter cloacae* complex was consistently identified, with annual counts ranging from 2 to 24 isolates and its highest annual proportion observed in 2022 (22.2%). Less frequently identified Enterobacterales species were grouped as “Other” and represented a variable proportion of annual CRE isolates.

## 4. Discussion

This study provides one of the few longitudinal evaluations of CRE from a Saudi secondary healthcare setting, describing eight-year trends in prevalence, species distribution, and antimicrobial resistance. Overall, CRE accounted for 28.8% of Enterobacterales isolates. Multivariable analysis demonstrated that the odds of CRE increased significantly over the study period and were independently associated with male sex and specimen source. Although prevalence rose steadily between 2017 and 2021, it declined thereafter while remaining substantially higher than in the early years of the study. The predominance of *K. pneumoniae* among CRE isolates and the high levels of resistance across multiple antimicrobial classes observed in this study highlight the substantial burden of CRE in hospital settings. These findings reinforce the importance of continuous local surveillance, as resistance epidemiology may change over time and differ considerably between healthcare institutions. They are also consistent with the growing global concern surrounding CRE as a major cause of healthcare-associated infections with limited therapeutic options and significant clinical impact [1,2].

The significant overall temporal increase in CRE prevalence observed in this study is consistent with reports from Saudi Arabia and other regions of the Middle East, where CRE prevalence has increased over the past decade. A recent systematic review and meta-analysis from Saudi Arabia reported pooled carbapenem resistance rates of 6.6% for imipenem, 9.1% for meropenem, and 18.6% for ertapenem among Enterobacterales, while also demonstrating considerable heterogeneity between healthcare institutions and geographic regions [6]. Similarly, surveillance from western Saudi Arabia documented increasing CRE prevalence across tertiary hospitals, highlighting substantial inter-facility variation despite the shared healthcare system [9]. Comparable observations have been reported across Gulf countries. A national surveillance study from the United Arab Emirates demonstrated changing CRE epidemiology over an 11-year period, with marked variation in prevalence and resistance patterns among participating hospitals [10]. Together, these findings indicate that although regional trends consistently demonstrate an increasing burden of CRE, local epidemiological surveillance remains essential because antimicrobial resistance patterns vary considerably between institutions, patient populations, and healthcare settings. The present study extends these observations by providing longitudinal data from a secondary healthcare hospital, a setting for which long-term surveillance data remain comparatively limited.

Several studies have reported that the COVID-19 pandemic was associated with increased antibiotic use, changes in healthcare utilization, and disruption of infection prevention practices, all of which may have influenced antimicrobial resistance trends in hospital settings [5,12]. International studies have shown that CRE epidemiology changed during the pandemic period, although the direction and magnitude of these changes differed between regions and healthcare systems. In the Southeast Asia region, Linn et al. reported changes in CRE incidence during the COVID-19 pandemic, emphasizing the importance of continued surveillance during periods of healthcare system disruption [13]. Similarly, a multicenter study from Italy comparing the pre-pandemic and first pandemic year demonstrated changes in colonization with carbapenem-resistant Enterobacterales, highlighting the potential impact of pandemic-related pressures on CRE transmission dynamics [14]. In the present study, CRE prevalence peaked in 2021 and declined thereafter; however, the reasons for this post-2021 decline cannot be determined from the available data. Potential explanations include changes in antimicrobial prescribing, infection prevention practices, patient characteristics, and healthcare utilization after the acute pandemic period. However, stewardship interventions, antimicrobial consumption, and infection-control metrics were not evaluated, and no causal relationship can be inferred. These findings reinforce the importance of sustained institutional surveillance to monitor CRE epidemiology during and after major healthcare disruptions.

Species distribution in our study was dominated by *K. pneumoniae*, which accounted for 50.1% of all CRE isolates, followed by *E. coli* (16.5%) and *Enterobacter cloacae* complex (14.8%). This distribution is comparable to reports from Saudi Arabia, where *K. pneumoniae* consistently represents the predominant CRE species, although the relative contribution of other Enterobacterales varies between institutions [6,8]. Similar findings have also been reported across Gulf countries, where *K. pneumoniae* has remained the principal CRE pathogen in hospital settings [5,7]. The predominance of *K. pneumoniae* is likely related to its ability to acquire and disseminate plasmid-mediated carbapenemase genes, particularly OXA-48-like and NDM enzymes, as well as its capacity for prolonged persistence and transmission within healthcare environments [5,11,15]. In contrast, *E. coli* and *Enterobacter cloacae* complex accounted for a smaller proportion of CRE isolates but remained consistently identified throughout the surveillance period, emphasizing that carbapenem resistance is not restricted to a single species. These findings support the need for continuous species-level surveillance because shifts in species distribution may influence both empirical antimicrobial selection and infection prevention strategies.

Beyond carbapenem resistance, CRE isolates demonstrated extensive non-susceptibility to several clinically important antimicrobial classes. Non-susceptibility to cefepime remained high throughout the study period, ranging from 78.3% in 2024 to 97.0% in 2021, while ceftazidime non-susceptibility ranged from 78.9% in 2017 to 96.9% in 2021. Fluoroquinolone non-susceptibility was particularly pronounced, with ciprofloxacin increasing from 78.9% in 2017 to 100% in both 2023 and 2024. These findings are consistent with previously reported CRE resistance profiles in Saudi Arabia and the broader Middle East, where resistance to cephalosporins and fluoroquinolones has been widely documented [6,7]. The high levels of non-susceptibility observed for these agents are clinically important because cephalosporins and fluoroquinolones are commonly considered in empirical or targeted treatment pathways for Gram-negative infections, and their reduced activity further limits available therapeutic options.

In contrast, aminoglycosides and tigecycline demonstrated comparatively lower non-susceptibility than cephalosporins and fluoroquinolones, although resistance remained substantial. Amikacin non-susceptibility ranged from 50.0% in 2019 to 73.1% in 2021, while gentamicin ranged from 59.3% in 2024 to 83.3% in 2022. Tigecycline demonstrated the lowest overall non-susceptibility among the antimicrobials evaluated, ranging from 26.6% in 2023 to 67.4% in 2019. Similar patterns have been reported in regional surveillance studies, where aminoglycosides and tigecycline often retain greater activity than cephalosporins and fluoroquinolones against CRE isolates [9]. Nevertheless, the resistance levels observed in this study indicate that these agents cannot be assumed to remain active and should be guided by isolate-specific susceptibility testing. Temporal trend analysis further showed that changes in non-susceptibility were antimicrobial-specific rather than uniform across drug classes, with significant trends observed for cefepime, ceftazidime, ciprofloxacin, and tigecycline.

From a clinical perspective, the resistance levels observed in this study highlight the therapeutic challenges associated with CRE infections. Non-susceptibility approached or exceeded 90% for several agents during specific years, including meropenem, cefepime, ceftazidime, and ciprofloxacin, indicating severely restricted activity of commonly used antimicrobial classes. Such resistance patterns complicate empirical treatment of severe Gram-negative infections, particularly when CRE is suspected before final susceptibility results are available. Although aminoglycosides and tigecycline demonstrated comparatively lower resistance than cephalosporins and fluoroquinolones, their activity was variable across years and species, reinforcing the need for isolate-specific susceptibility testing rather than reliance on class-level assumptions. These findings highlight the importance of institution-specific antibiograms and ongoing surveillance to inform empirical antimicrobial selection, optimize antimicrobial stewardship interventions, and support infection prevention programs. Reliance solely on national or regional surveillance data may overlook important local epidemiological differences that directly influence treatment decisions, particularly in hospitals with local resistance patterns that differ from multicenter averages.

This study has several strengths. It provides one of the longest longitudinal evaluations of CRE from a Saudi secondary healthcare setting, allowing evaluation of temporal trends in CRE prevalence, species distribution, and antimicrobial resistance patterns. The use of a patient-level deduplication strategy minimized bias associated with repeated isolation from the same individual and provided a more accurate estimate of annual resistance trends. In addition, the inclusion of species-level distribution data offers important insight into the local epidemiology of CRE in a secondary healthcare setting, where published longitudinal data remain limited. However, several limitations should be considered. First, this was a retrospective laboratory-based study conducted at a single center, which may limit the generalizability of the findings to other institutions. Additionally, the relatively small number of isolates in the early years should be considered when interpreting temporal trends. Second, the dataset did not distinguish between infection and colonization, as isolates were derived from routine clinical microbiology records. This limitation is particularly relevant for urinary and respiratory specimens, where positive cultures may represent colonization rather than true infection. Consequently, the reported CRE prevalence reflects microbiological isolate prevalence rather than the burden of clinically confirmed CRE infections. Accordingly, the present study was not designed to evaluate antimicrobial treatment decisions or their association with patient outcomes. Third, molecular characterization of the isolates was not performed; therefore, the distribution of carbapenemase genes, such as OXA-48, NDM, KPC, and VIM, could not be determined. Because these enzymes have important epidemiological relevance in Saudi Arabia and the Arabian Peninsula [7,8], the absence of molecular data limits interpretation of the underlying resistance mechanisms and comparisons with regional molecular surveillance studies. Finally, ertapenem susceptibility testing was not routinely performed during the study period; therefore, the definition of CRE was based on imipenem and meropenem non-susceptibility, which may have underestimated isolates with isolated ertapenem resistance. In addition, susceptibility results were interpreted according to the CLSI breakpoints in use at the time of testing. Consequently, breakpoint updates during the study period may have influenced temporal comparisons.

In conclusion, CRE represented a substantial proportion of Enterobacterales isolates in this Saudi secondary healthcare setting and demonstrated persistently high resistance to multiple clinically important antimicrobial classes. Although CRE prevalence declined after peaking in 2021, the overall temporal trend remained upward over the eight-year study period. These findings provide locally relevant evidence to support empirical antimicrobial prescribing, antimicrobial stewardship, and infection prevention strategies in similar healthcare settings.

## Figures and Tables

**Table 1:** Demographic characteristics and specimen sources of patients with Enterobacterales isolates.

Characteristic	*n* (%) *N* = 2378
Sex	
Male	1382 (58.1)
Female	996 (41.9)
Age	
0–18	45 (1.9)
19–30	254 (10.7)
31–45	498 (20.9)
46–60	375 (15.8)
>60	1206 (50.7)
Nationality	
Saudi	1843 (77.5)
Non-Saudi	535 (22.5)
Source of isolates	
Blood and sterile body fluids	300 (12.6)
Respiratory tract	539 (22.7)
Urine and genital tract	994 (41.8)
Wound, pus, and tissue	537 (22.6)
Stool and gastrointestinal tract	8 (0.3)
CRE status	
CRE isolates	684 (28.8)
Non-CRE isolates	1694 (71.2)

**Table 2:** Multivariable logistic regression analysis of factors associated with CRE.

Variable	Adjusted OR (95% CI)	*p* Value
Collection year (per year)	1.12 (1.08–1.17)	<0.0001
Sex		
Female	Reference	
Male	1.48 (1.21–1.81)	<0.0001
Age group (years)		
>60	Reference	
0–18	1.04 (0.55–1.96)	0.9120
19–30	0.82 (0.60–1.13)	0.2242
31–45	0.70 (0.54–0.90)	0.0056
46–60	0.82 (0.63–1.08)	0.1590
Source of isolates		
Blood and sterile body fluids	Reference	
Respiratory tract	2.05 (1.51–2.79)	<0.0001
Urine and genital tract	0.65 (0.49–0.88)	0.0048
Wound, pus, and tissue	0.86 (0.62–1.19)	0.3591

OR, odds ratio; CI, confidence interval. Stool and gastrointestinal tract specimens were excluded from the regression analysis because of the small number of isolates (n = 8). Odds ratios are adjusted for all variables included in the model. Age >60 years, female sex, and blood and sterile body fluid specimens were used as the reference categories.

**Table 3:** Annual antimicrobial non-susceptibility among CRE, 2017–2024.

Drug	2017	2018	2019	2020	2021	2022	2023	2024	*p* Value
Imipenem	21 (100)	61 (87.1)	43 (87.8)	90 (76.9)	101 (75.9)	93 (87.7)	68 (79.1)	75 (82.4)	0.5389
Meropenem	21 (100)	57 (81.4)	40 (80.0)	105 (88.2)	119 (90.8)	92 (88.5)	70 (82.4)	66 (72.5)	0.7057
Piperacillin/tazobactam	8 (50.0)	54 (75.0)	35 (70.0)	95 (79.8)	114 (85.7)	87 (80.6)	58 (67.4)	56 (61.5)	0.1772
Cefepime	14 (82.4)	63 (88.7)	42 (85.7)	101 (87.1)	129 (97.0)	100 (92.6)	74 (86.0)	72 (78.3)	0.0035
Ceftazidime	15 (78.9)	57 (91.9)	44 (88.0)	105 (92.1)	125 (96.9)	100 (93.5)	70 (92.1)	78 (89.7)	<0.0001
Amikacin	13 (65.0)	46 (63.0)	25 (50.0)	73 (61.9)	98 (73.1)	74 (68.5)	51 (58.6)	46 (50.5)	0.9492
Gentamicin	15 (71.4)	53 (72.6)	36 (72.0)	90 (75.6)	109 (81.3)	90 (83.3)	65 (74.7)	54 (59.3)	0.4260
Ciprofloxacin	15 (78.9)	62 (87.3)	41 (82.0)	104 (88.9)	125 (93.3)	102 (96.2)	87 (100)	90 (100)	<0.0001
Tigecycline	5 (41.7)	40 (63.5)	31 (67.4)	60 (53.1)	56 (46.3)	44 (43.1)	21 (26.6)	39 (44.8)	0.0063

Data are presented as n (%). Percentages were calculated using the number of CRE isolates tested against each antimicrobial agent in each year as the denominator. Non-susceptibility was defined as isolates categorized as intermediate or resistant. *p* values were derived from logistic regression using collection year as a continuous predictor.

**Table 4:** Annual species distribution of CRE, 2017–2024.

Species	2017 n = 21	2018 n = 73	2019 n = 50	2020 n = 119	2021 n = 134	2022 n = 108	2023 n = 87	2024 n = 92
*Klebsiella pneumoniae*	7 (33.3)	32 (43.8)	22 (44.0)	72 (60.5)	86 (64.2)	59 (54.6)	35 (40.2)	30 (32.6)
*Escherichia coli*	6 (28.6)	9 (12.3)	8 (16.0)	15 (12.6)	17 (12.7)	13 (12.0)	17 (19.5)	28 (30.4)
*Enterobacter cloacae*	2 (9.5)	11 (15.1)	6 (12.0)	16 (13.4)	16 (11.9)	24 (22.2)	13 (14.9)	13 (14.1)
Other	6 (28.6)	21 (28.8)	14 (28.0)	16 (13.4)	15 (11.2)	12 (11.1)	22 (25.3)	21 (22.8)

Data are presented as n (%). Percentages were calculated using the annual number of CRE isolates as the denominator. Only species with >30 isolates during the study period are shown; less frequent species were grouped as “Other”.

## Data Availability

The datasets generated and/or analyzed during the current study are not publicly available due to institutional restrictions but are available on reasonable request and with permission from the relevant institutional authorities.

## Supplementary Materials

The following supporting information can be downloaded at: https://www.mdpi.com/article/10.3390/microorganisms14081604/s1, Table S1: Annual antimicrobial non-susceptibility among the three predominant carbapenem-resistant Enterobacterales species, 2018–2024.

## Abbreviations

The following abbreviations are used in this manuscript:

CLSI	Clinical and Laboratory Standards Institute
CRE	Carbapenem-resistant Enterobacterales
CI	Confidence interval
OR	Odds ratio
