# Supplementary material for: Antimicrobial Resistance Trends in Carbapenem-Resistant Enterobacterales in a Saudi Healthcare Setting
Source: Microorganisms. 2026 Jul 23;14(8):1604. doi: 10.3390/microorganisms14081604 (PMC13515780; doi:10.3390/microorganisms14081604)
Supplement: Supplementary file 1 [file microorganisms-14-01604-s001.zip › microorganisms-4414132-supplementary.pdf]

Supplementary Table S1. Annual antimicrobial non-susceptibility among the three predominant carbapenem-resistant Enterobacterales species, 2018–2024.

**Species**	**Drug**	**2018**	**2019**	**2020**	**2021**	**2022**	**2023**	**2024**
Klebsiella pneumoniae	Imipenem	27 (84.4)	19 (86.4)	51 (70.8)	61 (70.9)	47 (81.0)	29 (82.9)	26 (86.7)
	Meropenem	26 (81.3)	20 (90.9)	71 (98.6)	81 (96.4)	54 (96.4)	31 (88.6)	26 (89.7)
	Piperacillin/tazobactam	25 (80.6)	18 (81.8)	67 (93.1)	79 (91.9)	53 (89.8)	28 (82.4)	22 (73.3)
	Cefepime	28 (90.3)	19 (86.4)	67 (94.4)	84 (98.8)	59 (100.0)	34 (97.1)	26 (86.7)
	Ceftazidime	22 (88.0)	20 (90.9)	71 (98.6)	83 (98.8)	58 (100.0)	28 (100.0)	25 (92.6)
	Amikacin	23 (71.9)	11 (50.0)	50 (69.4)	68 (79.1)	49 (83.1)	24 (68.6)	16 (53.3)
	Gentamicin	23 (71.9)	13 (59.1)	56 (77.8)	72 (83.7)	54 (91.5)	31 (88.6)	20 (66.7)
	Ciprofloxacin	24 (80.0)	18 (81.8)	69 (98.6)	83 (96.5)	55 (94.8)	35 (100.0)	29 (100.0)
	Tigecycline	15 (48.4)	17 (77.3)	37 (52.1)	31 (38.8)	20 (33.9)	7 (20.6)	14 (46.7)
Escherichia coli	Imipenem	7 (77.8)	7 (87.5)	12 (80.0)	14 (82.4)	13 (100.0)	12 (70.6)	21 (75.0)
	Meropenem	7 (87.5)	6 (75.0)	8 (53.3)	13 (76.5)	9 (69.2)	14 (82.4)	19 (67.9)
	Piperacillin/tazobactam	7 (77.8)	6 (75.0)	7 (46.7)	9 (56.3)	6 (46.2)	7 (41.2)	10 (35.7)
	Cefepime	9 (100.0)	7 (100.0)	9 (69.2)	16 (94.1)	10 (76.9)	14 (87.5)	22 (78.6)
	Ceftazidime	5 (100.0)	7 (87.5)	12 (85.7)	14 (93.3)	11 (84.6)	14 (93.3)	23 (85.2)
	Amikacin	5 (55.6)	4 (50.0)	5 (33.3)	10 (58.8)	4 (30.8)	10 (58.8)	16 (57.1)
	Gentamicin	6 (66.7)	7 (87.5)	9 (60.0)	14 (82.4)	6 (46.2)	12 (70.6)	15 (53.6)
	Ciprofloxacin	9 (100.0)	8 (100.0)	11 (73.3)	16 (94.1)	13 (100.0)	17 (100.0)	28 (100.0)
	Tigecycline	6 (75.0)	4 (57.1)	7 (46.7)	10 (62.5)	4 (33.3)	6 (40.0)	12 (44.4)
Enterobacter cloacae complex	Imipenem	11 (100.0)	5 (83.3)	14 (87.5)	14 (87.5)	23 (95.8)	10 (76.9)	10 (76.9)
	Meropenem	10 (90.9)	6 (100.0)	14 (87.5)	12 (80.0)	22 (91.7)	11 (84.6)	11 (84.6)
	Piperacillin/tazobactam	11 (100.0)	4 (66.7)	11 (68.8)	15 (93.8)	23 (95.8)	9 (69.2)	10 (76.9)
	Cefepime	10 (90.9)	5 (83.3)	13 (81.3)	15 (93.8)	24 (100.0)	11 (84.6)	12 (92.3)
	Ceftazidime	11 (100.0)	5 (83.3)	12 (80.0)	15 (93.8)	24 (100.0)	12 (92.3)	13 (100.0)
	Amikacin	9 (81.8)	5 (83.3)	10 (66.7)	10 (62.5)	16 (66.7)	5 (38.5)	8 (61.5)
	Gentamicin	9 (81.8)	5 (83.3)	11 (68.8)	12 (75.0)	22 (91.7)	10 (76.9)	9 (69.2)
	Ciprofloxacin	10 (90.9)	4 (66.7)	12 (75.0)	14 (87.5)	22 (95.7)	13 (100.0)	13 (100.0)
	Tigecycline	10 (100.0)	3 (60.0)	10 (62.5)	10 (66.7)	14 (66.7)	3 (25.0)	8 (61.5)

Data are presented as n (%). Percentages were calculated using the number of isolates tested against each antimicrobial agent for each species and year as the denominator. Non-susceptibility was defined as isolates categorized as intermediate or resistant according to CLSI interpretive criteria applicable at the time of testing. Data are presented from 2018 onward because of the limited number of isolates available for species-specific analysis in 2017.
